# The Impact of Music Perception on Quantitative Sensory Testing (QST)

**DOI:** 10.3390/jcm13092471

**Published:** 2024-04-24

**Authors:** Stefan Evers, Henning Brameyer, Esther Pogatzki-Zahn

**Affiliations:** 1 Department of Neurology, University of Münster, 48149 Münster, Germany; 2 Department of Neurology, Krankenhaus Lindenbrunn, 31863 Coppenbrügge, Germany; 3 Department of Anaesthesiology, University of Münster, 48149 Münster, Germany

**Keywords:** music, quantitative sensory testing, pain perception

## Abstract

**Objective**: The impact of listening to music on pain perception has been evaluated using questionnaires and numeric/visual analogue scales. In this study, the impact of music perception on sensory pain functions was measured by means of quantitative sensory testing. **Methods**: We enrolled 10 female and 10 male healthy subjects (10 of them were professional musicians). All subjects underwent, in total, four quantitative sensory testing measures (first: baseline; second: after pleasant music [Johannes Brahms, 3rd symphony, 3rd movement]; third: after unpleasant music [Krzysztof Penderecki, Threnos]; fourth: after a longer break). The pleasantness of music was evaluated using the Ertel differential scale. **Results**: After the participants listened to pleasant music, an increased sensitivity to cold stimuli (both threshold and pain), to mechanical stimuli (only for threshold), and to repeated stimuli (wind-up reaction) was noted. Listening to unpleasant music was not associated with changes in sensitivity. We did not observe any significant differences between male and female subjects or between musicians and non-musicians. There was no significant correlation between the rating of the music as pleasant/unpleasant and the different quantitative sensory testing measures. **Conclusions**: Our data show that listening to music inducing a pleasant feeling can increase the sensitivity to stimuli applied during a quantitative sensory testing session. This should be considered when performing or interpreting quantitative sensory testing examinations. Interestingly, this finding is in contrast to the observation that listening to music can decrease pain perception during painful procedures.

## 1. Introduction

Music in the treatment of pain has been applied already since antiquity. Even today, music is used as the only, or as an adjunctive, modality in some types of acute or chronic pain treatment [1,2,3]. The pain modulating effect of musical stimuli and the impact of music on other vegetative parameters are explained to date through the close anatomical localisation between the acoustic pathways and the brainstem and cerebral regions of pain perception, including the limbic system and vegetative nuclei [4,5,6,7,8]. Further, neuroendocrinological examinations have shown that music perception can influence the release of stress hormones and endorphins [9,10,11]. However, all these effects are rather small.

Different physiological parameters have been used to study the impact of music perception on physiological functions. Mainly, vegetative functions such as blood pressure, pulse frequency, breathing frequency, and sympathetic skin reflex have been assessed [12,13,14,15]. However, these parameters are quite unspecific in the evaluation of the physiological responses to music perception with respect to pain perception. Meanwhile, music perception is often recommended in the treatment of pain; music therapy is a part of multimodal pain therapy in some clinics. It is important to verify the effectiveness of listening to music through experimental measures in order to justify the usefulness of music in pain treatment.

Therefore, we aimed to measure the influence of music perception on physiological somatosensory functions with a semi-objective and quantitative method. We controlled these measures for the musical experience of the subjects and for the character of the music. We chose the standardised procedure of quantitative sensory testing (QST) which has been established and used to evaluate several submodalities of somatosensory processing, including different pain modalities as processed by different groups of nerve fibres (A-δ, A-β, C) [16,17,18].

Measurable changes in QST parameters induced by music perception could be useful in evaluating the most effective setting of music therapy for patients with acute or chronic pain.

## 2. Subjects and Methods

### 2.1. Subjects

We enrolled 20 healthy subjects (10 male and 10 female). They were divided into two groups: musicians (*n* = 10) and non-musicians (*n* = 10). Inclusion criteria for being a musician were at least two years of professional musical education or active membership in an orchestra or choir. Non-musicians were only allowed to have normal school education in music but no experience in playing an instrument. The mean age of all subjects was 23.1 +/− 2.2, and there were no significant differences between musicians and non-musicians with respect to age or sex. All subjects had to be free of any neurological or psychiatric disorder and were not allowed to smoke or to take any medication (in particular, no analgesic or sedating drugs within five days before the study date). Oral contraceptive drugs were allowed for female subjects who were in the first two days of menstruation; however, this was also an exclusion criterion. All subjects provided written informed consent. The study was approved by the institutional review board, and the methods were approved by the ethics committee of the local Faculty of Medicine.

### 2.2. Study Design

In total, four QST measures were performed. After a baseline QST measure, a five-minute piece of pleasant music was presented to the subjects (Johannes Brahms, 3rd Symphony, op. 90, 3rd movement), and then, another QST measure was performed directly after 15 min. After the second QST measure, a five-minute piece of unpleasant music was presented to the subjects (Krzysztof Penderecki, Threnos, 1960/61). Again, a QST measure was performed directly after the music. Finally, subjects had a break of five minutes without any acoustic input, and a final QST measure was performed. The music was always presented to the subjects at the same loudness level and through the same CD device with earphones. The order was not random.

After the two music presentations and after the break, the subjects were asked to rate their affective impression of the music and the break. We used the so-called “Eindrucks differential“ according to Ertel [19]. This is a measure used to rate situations as pleasant versus unpleasant using a score between −18 (very unpleasant) and 18 (very pleasant). All measures were recorded on one day, and the whole procedure took about 90 min per subject.

### 2.3. QST Measure

QST is a method used to evaluate different types of pain perception in a semiquantitative way. It is based on proband answers but calculates z-scores with respect to the general population. The QST measure was performed according to the standardised protocol of the German Research Network on Neuropathic Pain (DFNS) [18]. The site of stimulation was the right forearm. After each measure, the exact placement of the stimulation device was changed in order to avoid peripheral habituation. We applied the following measures, always in the same order, for all probands:

1. Thermal testing (Aδ and C fibres): We used a computer-controlled thermotester TSA 2001-II (company Medoc, Tel Aviv, Israel) and a thermode with a 30 cm^2^ contact area. We evaluated the cold detection threshold (CDT), the warm detection threshold (WTD), the cold pain threshold (CPT), and the heat pain threshold (HPT) by calculating the mean of three consecutive measurements. First, we measured the CDT and WDT, and then we measured the CPT and HPT. All thresholds were measured using a temperature stimulus with a decrease (or increase) of 1 °C/s. The starting temperature was 32 °C, and the stopping temperatures were 50 °C and 0 °C.

2. Mechanic detection threshold (MDT): The MDT (i.e., the function of Aβ fibres) was measured using calibrated von Frey filaments (Optihair2-set, Marstock Nervtest, Heidelberg, Germany) with an intensity ranging from 0.25 mN to 512 mN. The contact area had a diameter of about 0.5 mm. We calculated the geometric mean of five threshold evaluations in both ascending and descending stimulus intensities.

3. Mechanic pain threshold (MPT): The MPT (i.e., functions of Aδ fibres) was measured using a similar procedure as for the MDT. We used pinprick punctuate probes (MRC systems, Heidelberg, Germany) with a contact area of a 0.2 mm diameter and standardised stimulus intensities (8, 16, 32, 64, 128, 256, 512 mN). The probands were asked to differentiate between a sharp and a dull experience. We calculated the geometric mean of five threshold evaluations in both ascending and descending stimulus intensities.

4. Windup ratio (WUR): The WUR was measured based on a pinprick punctuate probe of 256 mN, as described for the MPT. The probands were asked to compare a single stimulus with a series of 10 stimuli (1 Hz frequency) of the same intensity and localisation. The pain was rated on a numeric analogue scale from 0 to 100. In total, five measures were performed to calculate the ratio of the mean of the single stimuli and the mean of the repetitive stimuli.

5. Vibration detection threshold (VDT): The VDT (i.e., the activation of Vater-Pacini bodies) was evaluated using a graded tuning fork with 64 Hz and 8/8 grading. The fork was placed on the distal ulna. The lowest disappearance threshold of three repetitive stimulations was used.

6. Pain pressure threshold (PPT): This final measure evaluating mechano-insensitive nociceptors and C fibres was performed with a pressure algometer (FDN200, Wagner Instruments, Greenwich, CT, USA). The contact area was 1 cm^2^ on the thenar muscles. The maximum force produced using this algometer was 2000 kPa. The pressure stimulation was slowly increasing with a ramp of 50 kPa/s. The arithmetic mean of three consecutive, increasing measures was calculated.

All measures were performed by the same investigator trained before in QST measures in a quiet room at the University Hospital of Münster (Germany) at the same time of day. In the beginning, the measure was demonstrated in an area of the contralateral arm, which was not used for the QST measures in the later session [20]. During the QST measure, the probands were asked to keep their eyes closed. Instructions for the following activities were described previously [18,21,22]. We used the z-transformation for direct comparisons between the different QST measures according to the following formula (see also [18]): Z-score = (X single Patient − mean probands)/SD probands. For the calculation of the mean z-score and the standard deviation of the probands, we used the first training data point of every QST measure at baseline. Further, the z-scores reflect the loss and gain of sensory functions (a score above 0 denotes a gain of sensory function, and a value lower than 0 denotes a loss of sensory function). A z-score higher and lower than 2 and −2 was regarded as pathological. We did not compare our results to those of the standardised normal values of the German consortium on neuropathic pain because these values were developed to classify patients with pain disorders. Furthermore, they are less reliable than internal control values used to detect minor differences.

### 2.4. Data Evaluation

Data were analysed with the statistical program SPSS (version 18.0) and were presented as the arithmetic mean with the standard deviation or as percentage. We used non-parametric tests for group comparisons and for correlation analysis. The significance level was set at *p* = 0.05.

First, we compared the results of the nine different QST measures at the four time points for the total group of probands. For statistical comparisons, we used the Friedman test with the Wilcoxon test as post hoc tests. In the second analysis, we compared the changes in the QST measures with respect to the type of music using the Wilcoxon test. Then, we calculated the differences between baseline and the other time points both for the nine different QST measures and for the Ertel score and compared these results between musicians and non-musicians using the Mann–Whitney U-Tests. For correlation analysis, we used the Spearman rank correlation coefficient.

## 3. Results

In Table 1, the raw data of the nine different QST measures are presented for the four different time points separately. We used this analysis to explore whether there were any habituation effects during the experimental procedure. Interestingly, there were no significant differences between the results at baseline or after the final break, suggesting that the procedure was robust and no relevant habituation effects occurred. The results after listening to the different types of music differed significantly from the baseline for the CDT, CPT, and WUR.

For a further evaluation of the impact of the different types of music on the QST measures, we used the z-transformation as described in the methods section. The results are presented in Table 2. For the CDT, MDT, and WUR, we detected a significant difference between two types of music. After listening to Brahms’ music, these parameters were more sensitive compared to the time after listening to Penderecki’s music. In Table 3, the results of the musicians versus non-musicians are shown. We did not find any significant differences in the QST measures between the two groups.

The pleasantness and unpleasantness of the music was scored according to Ertel (see Section 2). The data are presented in Table 4. There was no significant difference between musicians and non-musicians with respect to the scores of the two types of music and the final break. We only observed a trend indicating that listening to Brahms’ music was associated with a more pleasant Ertel score in the non-musicians than in the musicians. However, this trend did not influence the data of the QST measures. The scoring was quite equivocal within the two groups, confirming that the two pieces of music were regarded as pleasant/unpleasant by all subjects in nearly the same way.

Since there were no significant differences in the Ertel score between the subgroups of musicians and non-musicians, we finally analysed the correlation between the Ertel score and the QST data for the whole group. The results are shown in Table 5. We observed only one significant correlation for the CPT after listening to Brahms’ music. This means that a higher score for pleasantness was associated with a higher positive z-score for the CPT.

## 4. Discussion

The aim of this exploratory study was to evaluate whether it is possible to influence sensory and nociceptive thresholds in healthy subjects through listening to music. For the first time, we used the method of QST to study this influence of music in order to obtain more objective results than just by asking subjects about the effects of music on their sensory perception.

The main result is that listening to pleasant music (here: 3rd Symphony by Johannes Brahms) was associated with changes in the measures of the CDT, CPT, MDT, and WUR. To summarise, these measures were more sensitive (gain of function) after listening to such pleasant music. Listening to unpleasant music did not result in changes in the QST measures.

Some studies from nursing research have shown that both active and receptive music therapy can contribute to a reduction in pain severity, to improved pain coping, and to an improvement of comorbidities such as depression and anxiety [13,23,24,25,26]. In these studies, the type of music was relevant for the beneficial effect since music can cause both activation and relaxation depending on the character of the music [12,27,28]. Music with activating properties should have the following characteristics: high loudness, frequent changes in loudness, rapid tempo, frequent changes in tempo, a broad range of tone frequency, and a moderate to high grade of complexity. On the other hand, music with relaxing properties should have the following characteristics: silence, only a few changes in loudness, slow tempo, only a few changes in tempo, a small range of tone frequency, and a low grade of complexity [29,30].

Surprisingly, our results show that listening to pleasant (i.e., relaxing) music was associated with a higher sensitivity to painful or unpleasant stimuli than listening to unpleasant music. According to our scoring instruments, relaxation is one aspect of pleasant music. We assume that the QST results reflect a direct impact of music on the somatosensory system, whereas the global rating of music as relaxing and thus pain relieving reflects the impact of music on emotional functions. Another explanation could be that the perception of pleasant music result in an increased attention to any type of stimuli and may thus induce a facilitation of painful stimuli. Finally, a vasodilation induced by the emotional state due to the music could be the underlying mechanism for impaired sensory experience. The latter explanation would primarily be a peripheral mechanism rather than a central neurological mechanism.

Previous studies have suggested that musical experience and training have a relevant impact on the emotional reactions to and on the irritability of music [31,32,33,34]. We could not confirm these findings since there were no significant differences between the musicians and non-musicians in our study in terms of any of the parameters. Even the scoring of pleasantness/unpleasantness was not different between musicians and non-musicians; however, there was a trend toward a more pleasant feeling in the non-musicians after listening to Brahms. However, it would be interesting to perform this experiment in subjects with amusia.

In a recent study, listening to preferred music reduced subjective pain perception in an experimental pain model but did not influence the RIII component of the nociceptive blink reflex [35]. This can be interpreted to be in line with our results in that nociceptive measures such as QST or the nociceptive blink reflex are not improved through pleasant music although listening to this music induces a general decrease in subjective (emotional) pain perception.

In studies on QST, music, per se, was able to reduce measures of mechanical pain sensitivity in healthy subjects [36,37], particularly through singing [38]. A recent study with healthy subjects showed that participant-chosen favourite music can improve several aspects of nociceptive processing as measured through QST compared to white noise or relaxing music [39]. However, this study did not evaluate the emotional impact of the music. A similar study, however, without using the full QST battery, also showed that listening to preferred music during nociception leads to higher pain thresholds and lower perceived pain scores in comparison to disliked music [40]. Interestingly, individuals with fibromyalgia were more pain sensitive than control subjects without fibromyalgia while listening to their favourite music [41].

The scientific explanations of the effects of music on pain perception are mainly based on psychological and psychodynamic theories [4,42,43]. The emotional reactivity to exogenic stimuli is considered the most important factor for any effects of such stimuli [44]. Since the sensory qualities of pain and temperature (transmitted through the funiculus anterolateralis) as well as proprioception and tactile stimulation (transmitted through the funiculus posterior) could be influenced by music perception, we assume that the anatomical region of the link between music and pain perception must be located in the thalamus and in higher cortical areas. Since our results were independent from the musical experience and education of the subjects, we assume that the influence of music on pain perception is not controlled by higher cortical/cognitive functions and is not dependent on conscious mechanisms. However, it is probably only a limited effect in time with the exception of the impact on emotions. Neurophysiological effects caused by music, such as mechanical pain among others, seem to be affected by music only for some minutes. This can also be seen with other physiological parameters such as heart rate or breathing.

Our study has some limitations. The major limitation is the artificial setting of the measures, which does not reflect the setting in pain therapy. Further, the short duration of music listening in our experimental setting does not reflect reality in music therapy. Finally, it might be that patients with pain experience music in a different way than subjects without pain. Therefore, a replication of this study with patients with chronic pain is warranted.

## 5. Conclusions

In conclusion, we demonstrated that listening to music can have a modulating effect on some QST parameters. Therefore, the emotional state (here: feeling of pleasantness/unpleasantness induced by music) must be considered when interpreting the results of QST. However, the effects caused by music were rather small and did not affect all levels of QST. Further, the data suggest that listening to music might have an impact on pain perception on a very basic level without a difference between musicians and non-musicians. Interestingly, only pleasant music induced a higher sensitivity to nociceptive stimuli, whereas unpleasant music did not induce any changes in QST parameters.

## Figures and Tables

**Table 1:** Results of QST (raw data) at baseline, after listening to Brahms‘ music, after listening to Penderecki’s music, and after a break at the end of examination. Data is given as arithmetic mean and standard deviation. Statistical comparison between all measures was performed by Friedman-test, post-hoc statistical comparison between two groups was performed by Wilcoxon-test (ns denotes not significant). Bold figures denote statistical significance.

	Baseline	After Brahms	After Penderecki	Significance	(After Break)
cold detection	**−1** **.9 +/− 2.4**	**−1.4 +/− 0.7**	**−1.9 +/− 0.9**	***p* = 0.043 ^a^**	−1.9 +/− 1.0
warm detection	1.9 +/− 0.5	2.2 +/− 1.0	1.9 +/− 0.6	ns (*p* = 0.538)	2.1 +/− 0.7
cold pain	**19.1 +/− 8.1**	**22.2 +/− 7.2**	**21.7 +/− 7.3**	***p* = 0.023 ^b^**	20.7 +/− 7.1
heat pain	41.2 +/− 3.4	41.6 +/− 3.2	41.2 +/− 3.4	ns (*p* = 0.377)	41.0 +/− 2.8
mechanic detection	3.3 +/− 3.0	2.4 +/− 2.4 ^c^	3.3 +/− 2.6	ns (*p* = 0.165)	3.4 +/− 2.6
mechanic pain	30.3 +/− 38.2	33.9 +/− 39.2	28.1 +/− 24.5	ns (*p* = 0.311)	28.1 +/− 21.5
wind-up	**2.8 +/− 1.2**	**2.8 +/− 1.3**	**3.4 +/− 1.3**	***p* = 0.022 ^d^**	3.0 +/− 1.7
vibration detection	7.2 +/− 0.5	7.1 +/− 0.8	7.2 +/− 0.8	ns (*p* = 0.408)	7.3 +/− 0.9
pressure pain	4.4 +/− 1.3	4.3 +/− 1.3	4.2 +/− 1.3	ns (*p* = 0.428)	4.3 +/− 1.1

post-hoc testing: ^a^ *p* = 0.013 for “after Brahms” versus “after Penderecki”. ^b^ *p* = 0.021 for baseline versus “after Brahms”. ^c^ *p* = 0.038 for “after Brahms” versus “after Penderecki”. ^d^ *p* = 0.021 for baseline versus “after Penderecki” and *p* = 0.015 for “after Brahms” versus “after Penderecki”.

**Table 2:** Results of QST after listening to Brahms‘ music and after listening to Penderecki’s music presented as z-transformation. Data is given as arithmetic mean and standard deviation. Statistical comparison between all measures was performed by Wilcoxon-test (ns denotes not significant). Bold figures denote statistical significance.

	After Brahms	After Penderecki	Significance
CDT	**0.5 +/− 2.2**	**0.1 +/− 2.0**	***p* = 0.014**
WDT	0.6 +/− 1.8	−0.01 +/− 1.1	ns (*p* = 0.296)
CPT	0.4 +/− 0.7	0.3 +/− 0.7	ns (*p* = 0.563)
HPT	0.01 +/− 0.7	−0.1 +/− 0.6	ns (*p* = 0.601)
MDT	**−0.3 +/− 0.8**	**0.02 +/− 0.6**	***p* = 0.018**
MPT	0.3 +/− 1.3	−0.1 +/− 0.8	ns (*p* = 0.433)
WUR	**0.1 +/− 0.5**	**0.5 +/− 0.8**	***p* = 0.017**
VDT	−0.1 +/− 1.1	0.1 +/− 1.2	ns (*p* = 0.285)
PPT	−0.1 +/− 0.3	−0.1 +/− 0.3	ns (*p* = 0.469)

**Table 3:** Changes of QST parameters (baseline minus after listening to Brahms’ music and baseline minus after listening to Penderecki’s music) presented separately for musicians and non-musicians. Data is given as arithmetic mean and standard deviation. Statistical comparison between all measures was performed by Mann-Whitney-U-test.

	After Brahms		After Penderecki	
	Musicians	Non-Musicians	Musicians	Non-Musicians
cold detection	0.3 +/− 1.0	0.7 +/− 3.0 (*p* = 0.912)	−0.1 +/− 1.4	0.3 +/− 2.6 (*p* = 0.529)
warm detection	0.5 +/− 1.0	0.7 +/− 2.4 (*p* = 0.796)	0.1 +/− 1.3	−0.1 +/− 1.1 (*p* = 0.853)
cold pain	0.4 +/− 0.5	0.3 +/− 0.9 (*p* = 1.0)	0.4 +/− 0.6	0.3 +/− 0.9 (*p* = 0.796)
heat pain	−0.1 +/− 0.6	0.1 +/− 0.8 (*p* = 0.684)	−0.2 +/− 0.3	−0.1 +/− 0.7 (*p* = 0.971)
mechanic detection	−0.4 +/− 0.8	−0.2 +/− 0.9 (*p* = 0.684)	0.1 +/− 0.6	−0.1 +/− 0.6 (*p* = 0.739)
mechanic pain	0.4 +/− 0.6	0.2 +/− 1.8 (*p* = 0.280)	0.1 +/− 0.5	−0.2 +/− 1.0 (*p* = 0.631)
wind-up	0.2 +/− 0.5	−0.1 +/− 0.5 (*p* = 0.315)	0.4 +/− 0.8	0.6 +/− 0.9 (*p* = 0.529)
vibration detection	−0.3 +/− 1.2	0.1 +/− 1.0 (*p* = 0.853)	−0.1 +/− 1.4	0.2 +/− 1.1 (*p* = 0.393)
pressure pain	−0.1 +/− 0.3	−0.1 +/− 0.3 (*p* = 0.739)	−0.1 +/− 0.2	−0.1 +/− 0.4 (*p* = 0.971)

**Table 4:** Scoring of the two music listening sessions and the final break according to the Ertel score. The higher the score is the more pleasant is the feeling of the subject. Statistical comparison by Mann-Whitney-U-test (ns denotes not significant).

	Musicians	Non-Musicians	Significance
	(*n* = 10)	(*n* = 10)	
Listening to Brahms	2.5 +/− 4.0	6.9 +/− 5.6	ns (*p* = 0.063)
Listening to Penderecki	−0.4 +/− 4.5	−0.5 +/− 4.2	ns (*p* = 1.0)
After final break	1.2 +/− 2.6	0.4 +/− 2.8	ns (*p* = 0.631)

**Table 5:** Correlation between the Ertel score and the results of QST separately for the QST after listening to Brahms’ music und after listening to Penderecki’s music. The Spearman-rank-correlation coefficient is given. Bold figures denote statistical significance.

	After Listening to Brahms’ Music	After Listening to Penderecki’s Music
cold detection	r = −0.067 (*p* = 0.780)	r = 0.265 (*p* = 0.258)
warm detection	r = −0.009 (*p* = 0.968)	r = −0.073 (*p* = 0.758)
cold pain	**r = 0.4** **18 *p* = 0.017**	r = −0.070 (*p* = 0.771)
heat pain	r = −0.024 (*p* = 0.921)	r = 0.076 (*p* = 0.749)
mechanic detection	r = 0.115 (*p* = 0.629)	r = −0.210 (*p* = 0.374)
mechanic pain	r = −0.214 (*p* = 0.378)	r = −0.194 (*p* = 0.414)
wind-up	r = 0.005 (*p* = 0.985)	r = −0.372 (*p* = 0.106)
vibration detection	r = −0.176 (*p* = 0.458)	r = −0.01 (*p* = 0.685)
pressure pain	r = 0.188 (*p* = 0.427)	r = 0.159 (*p* = 0.504)

## Data Availability

The data presented in this study are available on reasonable request from the corresponding author.
